# Potentially Modifiable Factors Associated with Death of Infants and Children with Severe Pneumonia Routinely Managed in District Hospitals in Malawi

**DOI:** 10.1371/journal.pone.0133365

**Published:** 2015-08-03

**Authors:** Penelope M. Enarson, Robert P. Gie, Charles C. Mwansambo, Alfred E. Chalira, Norman N. Lufesi, Ellubey R. Maganga, Donald A. Enarson, Neil A. Cameron, Stephen M. Graham

**Affiliations:** 1 International Union Against Tuberculosis and Lung Disease, Paris, France; 2 Desmond Tutu TB Centre, Stellenbosch University, Tygerberg, South Africa; 3 Department of Paediatrics and Child Health, Faculty of Medicine and Health Sciences, University of Stellenbosch, Tygerberg, South Africa; 4 Ministry of Health, Lilongwe, Malawi; 5 UNICEF Malawi, Lilongwe, Malawi; 6 Division of Community Health, The Department of Interdisciplinary Sciences, Faculty of Medicine and Health Sciences, University of Stellenbosch, Tygerberg, South Africa; 7 Centre for International Child Health, University of Melbourne Department of Paediatrics and Murdoch Children’s Research Institute, Royal Children’s Hospital, Melbourne, Australia; Liverpool School of Tropical Medicine, UNITED KINGDOM

## Abstract

**Objective:**

To investigate recognised co-morbidities and clinical management associated with inpatient pneumonia mortality in Malawian district hospitals.

**Methods:**

Prospective cohort study, of patient records, carried out in Malawi between 1^st^ October 2000 and 30^th^ June 2003. The study included all children aged 0-59 months admitted to the paediatric wards in sixteen district hospitals throughout Malawi with severe and very severe pneumonia. We compared individual factors between those that survived (n = 14 076) and those that died (n = 1 633).

**Results:**

From logistic regression analysis, predictors of death in hospital, adjusted for age, sex and severity grade included comorbid conditions of meningitis (OR =2.49, 95% CI 1.50-4.15), malnutrition (OR =2.37, 95% CI 1.94-2.88) and severe anaemia (OR =1.41, 95% CI 1.03-1.92). Requiring supplementary oxygen (OR =2.16, 95% CI 1.85-2.51) and intravenous fluids (OR =3.02, 95% CI 2.13-4.28) were associated with death while blood transfusion was no longer significant (OR =1.10, 95% CI 0.77-1.57) when the model included severe anaemia.

**Conclusions:**

This study identified a number of challenges to improve outcome for Malawian infants and children hospitalised with pneumonia. These included improved assessment of co-morbidities and more rigorous application of standard case management.

## Introduction

Pneumonia is consistently estimated to be the single major cause of death in infants and young children (1–59 months of age) and almost all these deaths occur in low-income countries. [1–3] It is estimated that approximately 50% of all deaths due to pneumonia in children occur in sub-Saharan Africa. [4] However, there are a number of acknowledged limitations to attributing a death to a single disease entity. [5–7] First, in infants and young children with severe pneumonia, co-morbidities such as malnutrition or HIV infection are common and increase the risk of death. [8,9] Second, there is clinical overlap with diseases such as severe anaemia, malaria or septicaemia that may result in death being wrongly attributed to pneumonia. [10,11] Finally, it is also recognised that bacterial pneumonia can occur as a co-infection or secondary complication in children with other infections such as measles, severe malaria or tuberculosis. [12–14] Nonetheless, health workers in high mortality settings are required to manage sick children according to standard case-management protocols on the basis of clinical findings with very limited diagnostic support.

The World Health Organization (WHO) has clear clinical case-management guidelines that aim to identify the child with pneumonia among the many infants and young children that present to health services with an acute respiratory illness, and then to classify the pneumonia case further by age and by severity. [15] These classifications determine whether the child with pneumonia is managed as an inpatient or outpatient and the choice of antibiotic treatment. [11] In general terms, the choice of penicillin is primarily aimed to treat pneumonia due to *Streptococcus pneumoniae*, while a broad spectrum antibiotic is recommended as first-line antibiotic treatment for cases where pneumonia is associated with a high risk of mortality and/or is often due to a wider range of bacterial pathogens, including Gram negative bacteria. The latter group include very young infants (<2 months of age), malnourished children and children with very severe pneumonia. [11,15] Therefore children 2 to 59 months of age classified as having very severe pneumonia are to be given chloramphenicol for treatment; those classified as having severe pneumonia are not. The WHO case-management guidelines also aim to avoid the unnecessary use of antibiotics for upper respiratory tract illness only.

We recently reported outcomes from a prospective implementation programme that included 47,228 Malawian children admitted to district hospitals in Malawi for severe and very severe pneumonia over a five year period. [16, 17] We have further analysed data from a sub-set of this cohort to determine the individual factors including demographics of the study population, recognised co-morbidities and clinical management that were associated with inpatient death.

## Methods

### Study participants

We reviewed the sequential records of all infants and young children (< 5 years) that were admitted to 16 district hospitals in Malawi between 1^st^ October 2000 and 30^th^ June 2003 as part of the Malawi Child Lung Health Programme (CLHP). The Malawi CLHP included a prospective evaluation of the implementation of standard case-management for 47,228 children admitted with severe or very severe pneumonia to one of the 24 district hospitals in Malawi over a 5-year period (2000–2005).

The implementation, methodology, evaluation and main outcomes of the Malawi CLHP have been described in detail in previous publications. [16, 17] Briefly, all neonates, infants and young children between 1 week and 59 months of age hospitalised with a clinical diagnosis of severe or very severe pneumonia, according to WHO definitions at the time, were included. The implementation followed training of staff to follow the recommended WHO pneumonia standard case management approach at the time of the study (Table 1) and it was ensured that supplies of treatment for child pneumonia (i.e. recommended antibiotics and oxygen therapy) were available. Oxygen was not available in all hospitals for the period of the study’s data collection. The first five district hospitals who started implementing the CLHP in October 2000 received their concentrators in February 2002. The remaining 11 received their concentrators in February 2003.

**Table 1:** WHO standard case management of pneumonia defined by age groups and severity of disease.

*Standard Case Management*		
*Diagnosis*	*Presenting signs and symptoms*	*Recommended treatment regimens*
***Child 2–59 months***		
***Severe pneumonia***	*Respiratory rate (bpm)*:	***Penicillin*** *50 000 units/kg 6 hourly for 3*
	*≥ 50 aged 2–11 months*	*days if improved then oral* ***amoxicillin*** *25*
	*≥ 40 aged 12–59 months*	*mg/kg three times daily for total of 5 to 8*
	*Lower chest wall indrawing*	*days*
***Very severe***	*Respiratory rate (bpm)*:	
***pneumonia***	*≥ 50 aged 2–11 months*	***Chloramphenicol*** *25 mg/kg 8 hourly for 5*
	*≥ 40 aged 12–59 months*	*days if improved then three times daily for*
	*Lower chest wall indrawing*	*total of 10 days antibiotic treatment*
	*Cyanosis*	
	*Unable to drink*	
	*Reduced level of consciousness*	
	*Grunting (infants)*	
***Infant <2 months***		
***Severe/Very severe***	*Respiratory rate (bpm)*:	***Gentamicin*** *7*.*5 mg/kg once daily for 8 days*
***pneumonia***	*≥ 60*	***Penicillin*** *50 000units/kg 6 hourly for three*
		*days if improved then oral* ***amoxicillin*** *25*
		*mg/kg three times daily for a total of 8 days*
		*antibiotic treatment*
***Co-morbid conditions/pneumonia treatment***		
***Pneumonia in***	*Signs and symptoms for severe*	***Cotrimoxazole*** *prophylaxis on admission if*
***severely***	*/very severe pneumonia as above*.	*not acutely ill*
***malnourished child***	*PLUS signs and symptoms for any*	*Treatment for severe or very severe*
	*of the following*:	*pneumonia as above PLUS*
	• *Marasmus*	***Gentamicin*** *(7*.*5 mg/kg IM/IV) once daily for*
	• *Kwashiorkor*	*7 days*
	• *<60% Weight for Height*	*➤If the child fails to improve within 48 hours*,
		*add* ***Chloramphenicol*** *(25 mg/kg IM/IV 8-*
		*hourly) for 5 days*.
***Known/suspected***	*2–6-month-old child with central*	***Continue first-line antibiotic (*** *such as*
***PcP***	*cyanosis*	***Chloramphenicol)*** *as mixed infection with*
	*Hyper-expanded chest*	*bacteria occurs*
	*Fast breathing*	***Oral Cotrimoxazole*:** *120mg three times*
	*Chest X-ray changes*, *but chest*	*daily if less than 5 kg; 240 mg three times*
	*clear on auscultation*	*daily if 5 kg or more for 21 days*
	*Enlarged liver*, *spleen*, *lymph*	
	*nodes*	
	*HIV test positive in mother or child*	

### Study procedures

All children included in the Malawi CLHP were allocated a “Pneumonia Inpatient Recording Form” which prospectively collected relevant data including demographic data, clinical data including weight, classification of severity of pneumonia, detailed data of management, and outcome data. In addition to training on the WHO standardised case-management approach to child pneumonia, staff were also trained on the diagnosis and management (according to national guidelines) of other related causes of common childhood illness such as malaria, anaemia or malnutrition.

The diagnosis and investigation of co-morbidities depended on clinical suspicion and usually clinical diagnosis. In addition, a thick blood film was examined for *Plasmodium falciparum* malaria diagnosis, or a haematocrit or haemoglobin were determined for diagnosis of anaemia. Chest radiography was not carried out routinely on admission but was used in those cases not responding to first-line antibiotics. No diagnostic tools were available or used to determine aetiological pathogen(s) causing pneumonia such as bacteria, viruses or fungi such as *Pneumocystis jirovecii*. Testing for HIV infection was rarely undertaken at the time of this study.

Weight was plotted for age on the *Road to Health Card*, and severe malnutrition was diagnosed if there was severe wasting or nutritional oedema. Severe malaria was diagnosed clinically in children with altered consciousness and/or convulsions

### Data management

Data from the Inpatient Recording Forms were double entered into an ACCESS database, and then transferred to EXCEL for cleaning. The following variables were selected for analysis: age, sex, classification of severity of pneumonia, any recorded comorbid conditions (included malaria, malnutrition, severe anaemia, meningitis and sepsis), origin of referral (self or transfer from a primary health care centre), antibiotic use prior to admission, and treatment given. See Table 2 for frequency of variables.

**Table 2:** Distribution of variables and CFR of consecutive children (0–59 months) treated for pneumonia in district hospitals in Malawi, 2000–2003.

Variable	Category	Number of cases	% of total	95% confidence interval		Number of deaths	CFR %
				lower	upper		
**Severity grade**	Very severe	5015	32.0	31.2	32.7	1,100	21.9
** **	Severe	10694	68.0	67.3	68.8	533	5.0
**Management**							
Type of referral	Self	12062	76.8	76.1	77.4	1146	9.5
	HC	2441	15.5	14.9	16.1	338	13.8
	Unknown	1206	7.7	7.39	8.1		
Antibiotic prior to admission	No	12288	78.2	77.6	78.9	1244	10.1
	Yes	3421	21.8	21.1	22.4	389	11.4
**Comorbidity**							
Malaria	Yes	8,013	51.0	50.2	51.8	768	9.6
	No	7,696	49.0	48.2	49.8	865	11.2
Malnutrition	Yes	826	5.3	4.92	5.62	192	23.2
	No	14,883	94.7	94.4	95.1	1441	9.7
Severe anaemia	Yes	621	4.0	3.76	4.36	113	18.2
	No	15,088	96	95.7	96.3	1520	10.1
Sepsis	Yes	99	0.6	0.51	0.76	16	16.2
	No	15,610	99.4	99.2	99.5	1617	10.4
Meningitis	Yes	93	0.6	0.48	0.72	33	35.5
** **	No	15,616	99.4	99.3	99.5	1600	10.2
**Additional treatment**							
Oxygen	Yes	1954	12.4	11.9	12.9	556	28.5
	No	13754	87.6	87.0	88.0	1077	7.8
	Unknown	1	0	0	0		
IV fluids	Yes	191	1.2	1.05	1.4	79	41.4
	No	15518	98.8	98.6	98.9	1554	10.0
Antimalarials	Yes	6288	40.0	39.3	40.8	538	8.6
	No	9420	60.0	59.2	60.7	1095	11.6
	Unknown	1	0	0	0		
Blood transfusion	Yes	494	3.1	2.9	3.43	83	16.8
** **	No	15215	96.9	96.6	97.1	1550	10.2

CFR: case-fatality rate; HC: Health Centre

### Statistical Analysis

Data were analysed using SPSS, version 7.5 for Windows and web-based OpenEpi (version 2.3). Because of the large sample size an effect size was arbitrary set at <0.67 and >1.5 to determine the importance of the odds ratio (OR). A probability level of <0.05 was considered significant and was used for inference.

Logistic regression analysis was performed to determine the association between variables by estimating the OR and 95% Confidence Intervals (CI) were calculated. Logistic regression was run to measure the independent effects of the significant variables on outcome—the proportion of the children who died while in hospital or case-fatality rate (CFR) was the primary outcome indicator measured. The OR and 95% CI were calculated to estimate the risk adjusting for potential confounders.

### Ethical considerations

The CLHP was standard practice of patient management and data from it was routinely collected. Patient identifiers such as names, geographic location by sub-district, patient registration number and specific date (except month and year) of admission were not included in database. Permission to use the data was received from the Ministry of Health, Republic of Malawi. Approval to analyze the data was obtained from the International Union Against Tuberculosis and Lung Disease (The Union) Ethical Advisory Group (EAG: 05/10) and the Human Research Ethics Committee (N10/09/285), Faculty of Medicine and Health Sciences, Stellenbosch University.

## Results

It was originally proposed that all information contained on each of the Inpatient Pneumonia Recording Forms would be entered into a computer data-base program by the CLHP Coordinators at each district. However, due to a larger-than-expected number of patients being admitted it was not possible for the Coordinators to undertake this task routinely. In 2003 The Union provided limited funding to the CLHP to enable it to contract a private data entry firm.

Between October 1st 2000 and June 30^th^ 2003 there were 17,480 (99.4%, 95% CI 99.3–99.5) Inpatient Pneumonia Recording Forms available for analysis from the 16 districts that had implemented the project for one or more years. From these, 837 were not eligible for the study (duplicate entries, retreatment following failure and non-severe pneumonia category), leaving a total of 16,643 eligible patients. Of them, 934 (5.3%, 95% CI 5.0–5.7) were excluded as their records contained insufficient data– 424 missing classification and 510 missing outcome; 15,709 children were included in the final analysis for this study.

**Fig 1:**
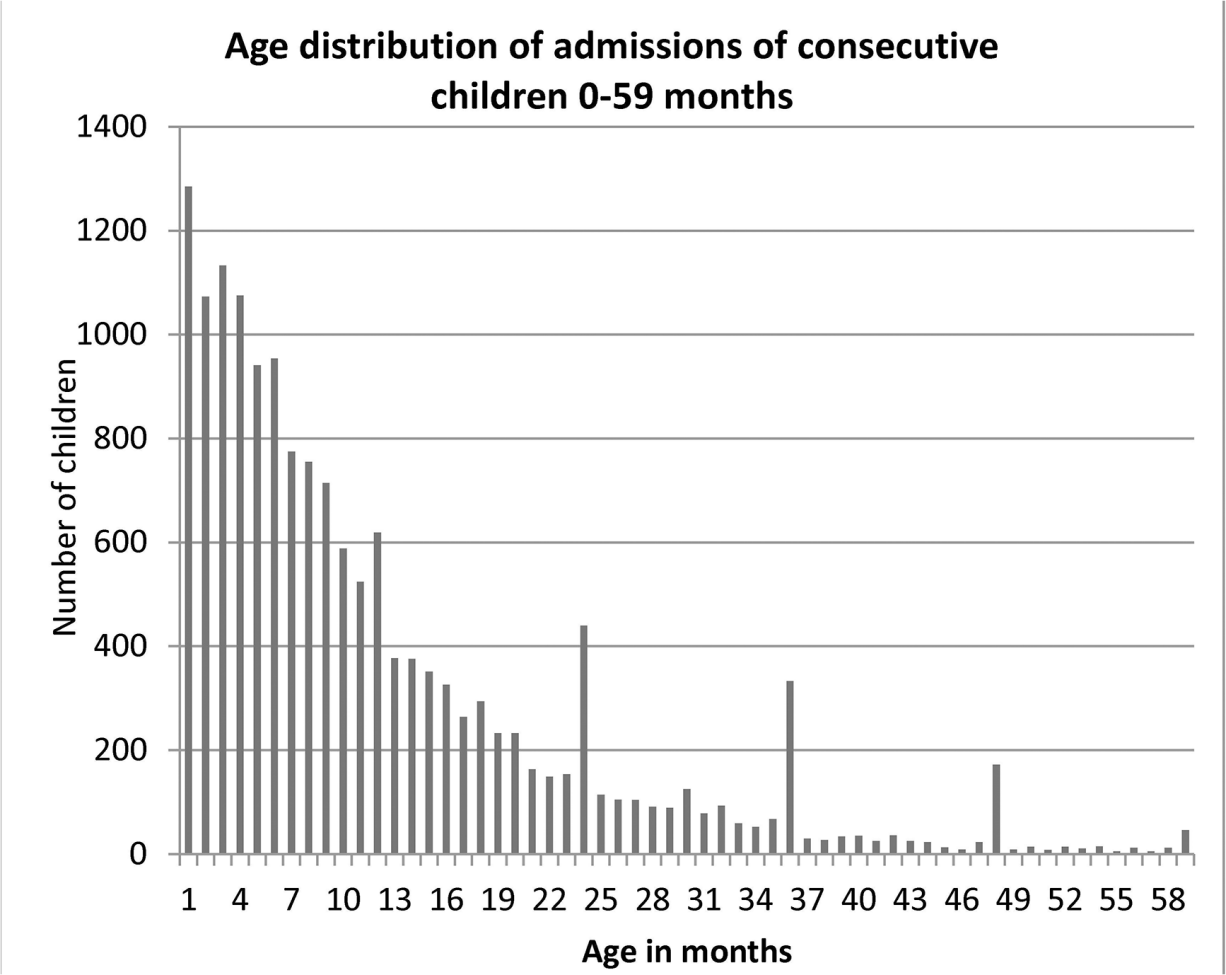
Age distribution of consecutive children (0–59 months) treated for pneumonia in district hospitals in Malawi, 2000–2003.

Fig 1 shows the age distribution of the 15,709 children included this group and provides a classic example of terminal digit preference, with ages 24, 36 and 48 months having a higher frequency than those immediately preceding and following them.

**Fig 2:**
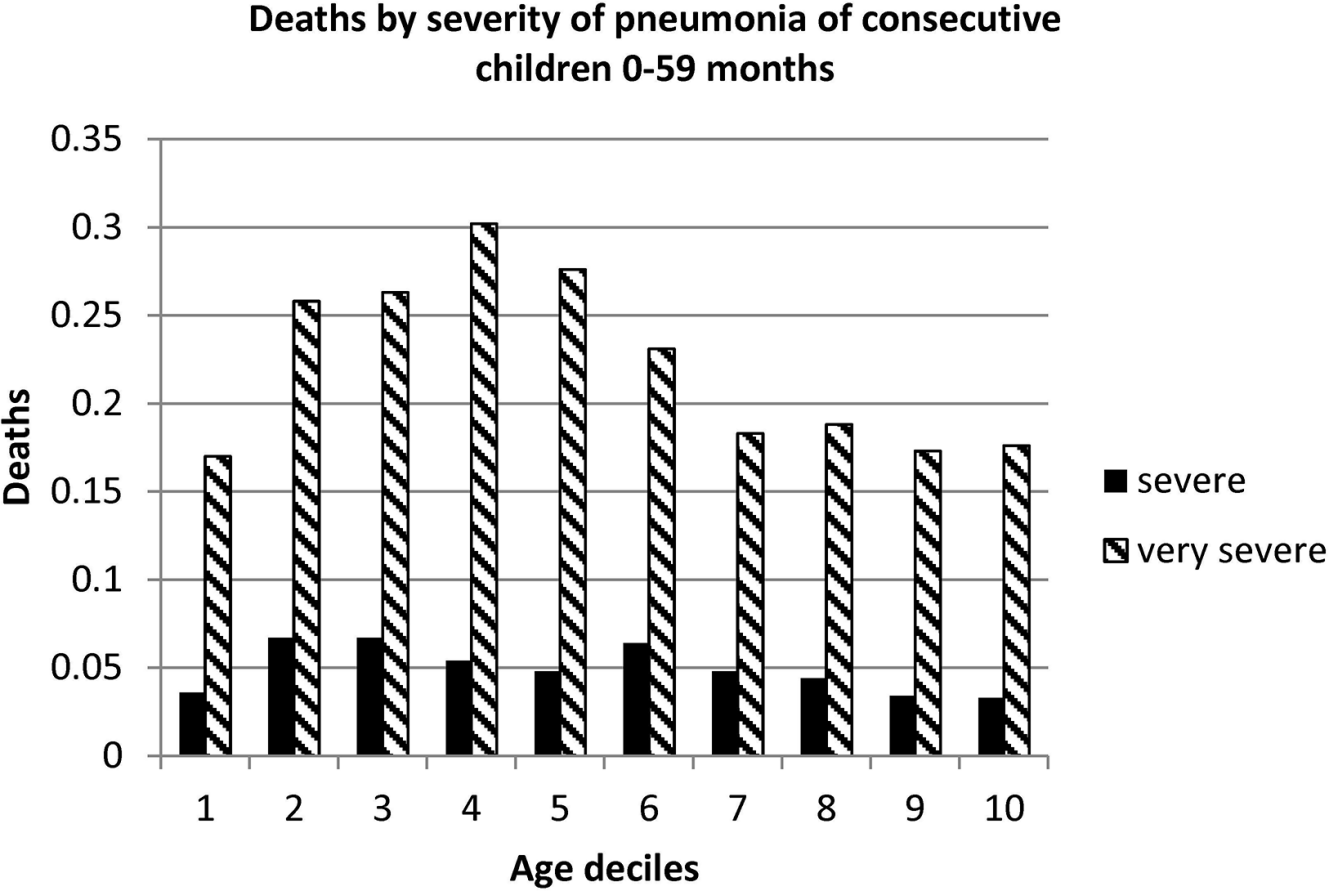
Case fatality rate of consecutive children (0–59 months) treated for pneumonia in district hospitals in Malawi, 2000–2003 by severity grade. For ease of comparison the children were sorted by age and then divided into ten equal groups (deciles). Consequently each of the ten deciles contains an equal number of children.

Table 2 lists the distribution and characteristics of variables and their associated CFR. There were 1,633 deaths with an overall CFR of 10.4% (95% CI 9.9–10.9). Very severe pneumonia was associated with a higher CFR than those classified as severe pneumonia **(**CRF 21.9%, 95% CI 20.8–23.1, as compared with 5.0%, 95% CI 4.6–5.4). This was consistent across the range of age (Fig 2). The majority (76.8%, 95% CI 76.1–77.4) of admissions were self-referred and these children were less likely to have received an antibiotic prior to admission (as compared to those referred from a primary health care centre).

Comorbid conditions were present on admission in 6,541 (61.2%, 95% CI 60.3–62.1) of severe pneumonia cases and in 2,806 (56.5%, 95% CI 55.1–57.9) of very severe pneumonia cases. Malaria was the most frequent comorbid condition diagnosed in 8,013 (51.0%, 95% CI 50.2–51.8) of all admitted cases.

The most frequent additional treatment modalities received were antimalarials 6,288 (40.0%, 95% CI 39.3–40.8) or oxygen therapy 1,954 (12.4%, 95% CI 11.9–12.9).

Table 3 shows crude and adjusted odds ratios and their 95% CI for the variables studied.

The highest adjusted odds ratio for death was for those children with very severe pneumonia (OR 4.05, 95% CI 3.53–4.64).

**Table 3:** Crude and adjusted odds ratios from logistic regression for mortality on management and comorbidity covariates adjusted for age, sex and severity grade of consecutive children (0–59 months) treated for pneumonia in district hospitals in Malawi, 2000–2003.

	Crude			Adjusted		
Variable	OR	95% CI		OR	95% CI	
		lower	upper		lower	upper
**Very severe vs severe pneumonia**	5.36	4.80	5.98	*4*.*05*	*3*.*53*	*4*.*64*
**Self, vs health centre, referral**	0.65	0.57	0.74	*1*.*32*	*1*.*13*	*1*.*55*
**Antibiotic prior to admission**	0.88	0.78	0.99	*0*.*99*	*0*.*85*	*1*.*14*
**Malaria**	0.84	0.76	0.93	*1*.*07*	*0*.*92*	*1*.*24*
**Malnutrition**	2.83	2.38	3.35	*2*.*37*	*1*.*94*	*2*.*88*
**Severe anaemia**	1.99	1.61	2.45	*1*.*41*	*1*.*03*	*1*.*92*
**Sepsis**	1.67	0.97	2.86	*1*.*07*	*0*.*32*	*3*.*59*
**Meningitis**	4.82	3.14	7.39	*2*.*49*	*1*.*50*	*4*.*15*
**Oxygen**	4.68	4.17	5.26	*2*.*16*	*1*.*85*	*2*.*51*
**IV fluids**	6.43	4.73	8.49	*3*.*02*	*2*.*13*	*4*.*28*
**Antimalarials**	0.71	0.62	0.79	*0*.*65*	*0*.*56*	*0*.*76*
**Blood transfusion**	1.78	1.39	2.27	*1*.*10*	*0*.*77*	*1*.*57*

Self-referral was associated with a significantly higher CFR while receiving an antibiotic prior to being referred to hospital was not.

Children had a significantly higher CFR with co-morbid conditions of meningitis (OR 2.49, 95% CI 1.50–4.15), malnutrition (OR 2.37, 95% CI 1.94–2.88) or severe anaemia (OR 1.41, 95% CI 1.03–1.92).

Children were more likely to die if they required supplementary oxygen (OR 2.16, 95% CI 1.85–2.51) or intravenous fluids (OR 3.02, 95% CI 2.13–4.28). Children treated with antimalarials were significantly less likely to die (OR 0.65, 95% CI 0.56–0.76). Blood transfusion was no longer significantly associated with CFR when adjusted for the other co-variants.

Of the 1,633 deaths 847 (51.9%, 95% Ci 49.4–54.3) died within 24 hours of admission. The determinants that were significantly associated with all deaths were re-evaluated to determine their association with time of death (within or beyond 24 hours). The one factor significantly more likely to be associated with death within 24 hours was the classification of very severe pneumonia (OR = 4.60, 95% CI 3.88–5.46 compared with OR = 2.92, 95% CI 2.47–3.45). Those dying after 24 hours of admission were significantly more likely to have comorbidity of meningitis (OR = 4.67, 95% CI 2.81–7.76 compared with OR = 1.17, 95% CI 0.54–2.54). The difference between those dying within 24 hours and those dying later was not statistically significant for age, malaria, malnutrition, severe anemia, oxygen therapy, IV fluids or antimalarials.

The patient disease classification was recorded for children over 2 months of age as it was written in the Inpatient Recording Forms. Each record also contained information on clinical symptoms and signs according to the routine practice of standard case management (Table 1). From these symptoms and signs, a post hoc classification was developed according to the definitions used in standard case management. Moreover, children with very severe pneumonia were recommended to receive chloramphenicol as part of their treatment, according to standard case management.

Table 4 presents the comparison for those children over 2 months of age between the recorded classification of severity of disease with the reclassification based on the recorded clinical details and whether or not the case was recorded as having been given chloramphenicol as part of treatment. The lowest case fatality ratio was in those recorded and reclassified as having severe pneumonia and in whom the treatment record did not include chloramphenicol (4.3%). It was highest in children who were treated with chloramphenicol in whom 1) classification was very severe pneumonia but reclassification was severe pneumonia (26.5%), 2) classification was severe pneumonia and reclassification was very severe pneumonia (25.2%) and 3) both classification and reclassification indicated very severe pneumonia (21.3%). In all instances CFR was higher when chloramphenicol was given than when it wasn’t regardless of concordance of classification / reclassification.

**Table 4:** Concordance of recorded and reclassified severity of disease, recorded antibiotic treatment and death while on treatment for children greater than 2 months of age in children treated for pneumonia in district hospitals in Malawi, 2000–2003.

Classification		Antibiotic	Total		Died			
Recorded	Reclassified	Chloramphenicol			No		Yes	
on admission		prescribed on admission	n =	%	n =	%	n =	%
All			14,047	100.0	12,599	89.7	1,448	10.3
Severe	Severe	no	6,865	100.0	6,570	95.7	295	4.3
Severe	Severe	yes	183	100.0	154	84.2	29	15.8
Severe	Very severe	no	2,794	100.0	2,647	94.7	147	5.3
Severe	Very severe	yes	127	100.0	95	74.8	32	25.2
Very severe	Severe	no	86	100.0	72	83.7	14	16.3
Very severe	Severe	yes	1,558	100.0	1,145	73.5	413	26.5
Very severe	Very severe	no	96	100.0	77	80.2	19	19.8
Very severe	Very severe	yes	2,338	100.0	1,839	78.7	499	21.3

## Discussion

We found a high mortality in a large cohort of 15,709 Malawian infants and children with severe pneumonia managed in 16 district hospitals. The CFR is similar to that previously reported for an even larger cohort of which this group reported here represent a sub-set. [17] The majority of cases presented to the hospital directly.

The commonest co-morbidity was malaria which was diagnosed in half of the cases. Features associated with an increased pneumonia-related mortality were the severity of pneumonia and the presence of co-morbidities such as malnutrition, severe anaemia or meningitis. While none of these associations is unexpected, the unique characteristic of the study is that these features have been analysed following prospective and complete clinical data collection from children managed under routine conditions at a district hospital level in a high mortality setting.

While data from a routine setting is a strength of the study, there are a number of important limitations that must be acknowledged. A major limitation of our study was that HIV prevalence data were not available for this cohort. While health workers were trained to recognise and treat *Pneumocystis jirovecii* pneumonia (PcP) and other HIV-related lung disease, this almost certainly did not occur as almost all participants’ HIV status was recorded as unknown. The lack of testing reflected both a lack of policy of routine testing in children with severe pneumonia, and a lack of willingness by health workers to undertake testing due to stigma compounded by a lack of access to anti-retroviral therapy at the time of the study.

However, the potential importance of HIV as a common co-morbidity in Malawian infants and children with severe pneumonia that had an impact on aetiology and outcome is recognised from studies reported from urban-based tertiary hospitals in Malawi and the region. [9] PcP is a common and often fatal cause of severe or very severe pneumonia in HIV-infected Malawian infants, especially between 2 and 6 months of age, and is likely to have contributed to the age-related mortality that was noted (Fig 2). [18,19] Clinical overlap with other pathogens, such as *Mycobacterium tuberculosis* and non-typhoidal Salmonella, both common in Malawi, may also have lessened the beneficial effect of the intervention as they would not be responsive to standard first-line antibiotic therapy. This issue was addressed more extensively in previously related article of the larger study group. [17]

A second limitation is that classification of severity grade, and therefore of “correct versus incorrect” antibiotic usage assumes that clinical signs and symptoms recorded on the Inpatient Pneumonia Recording Forms were accurate, but there is no way of verifying if this was the case. The decision to prescribe more powerful antibiotics was most closely associated with the likelihood of children dying. This suggests that a more accurate overall assessment of severity, as opposed to the severity category recorded in the case file, was used in the decision to use a more powerful antibiotic. At least the choice of antibiotic was not affected by a lack of availability in this study as implementation of the CLHP ensured availability of all antibiotics and no stock-outs. [16,17]

We were surprised to discover the extent of misclassification in this group of patients as we had previously concluded that standard case management was an important approach to reduce case fatality in this setting. We were unable to judge the extent of misclassification in our previous study [17] because we reported only aggregate data from routine reports. It was only when we analysed individual data in this part of the study that we were able to determine the extent of the misclassification, hence justifying the importance of analysing individual data in addition to reporting the results from analysis of aggregate data.

In the last decade the Ministry of Health have introduced a number of strategies that will have impacted on today’s pneumonia rates, care and outcomes. In 2005 based on results showing the efficacy of the use of cotrimoxazole preventive therapy (CPT) in the reduction of mortality and morbidity from opportunistic infections in HIV positive adults and children the Ministry of Health introduced a general policy on its use in Malawi. This included all children born to an HIV-positive woman aged 6 weeks or more and that they should receive CPT until HIV is excluded. Also included in the policy was that any HIV-positive child 6 weeks or above irrespective of symptoms, needs CPT prophylaxis for life. [20–22]

In July 2011, the Malawi Ministry of Health implemented ‘Option B+’ nationally, a policy to initiate ART for life, regardless of CD4 count, for all HIV-positive pregnant or breastfeeding women. Babies born to HIV positive mothers should also receive 6 weeks of nevirapine, PCR-based HIV screening at 6 weeks, additional rapid testing at 12 months and 24 months and start on ART if HIV-positive. [23–25]. These improved HIV/AIDS services in the country would be expected to also improve outcome of treatment of children with pneumonia.

Another limitation to the study is that the diagnosis of other co-morbidities was usually clinical without laboratory confirmation such as for malaria, sepsis or severe anaemia. Therefore, it cannot be determined whether these conditions were under-diagnosed or over-diagnosed, especially as there is clinical overlap with severe pneumonia. [26] Further, while recognised as co-morbidities that can influence outcome, the evidence of association for this study is weakened by the lack of laboratory confirmation. Similarly, malnutrition is a major recognised co-morbidity that increases pneumonia incidence and worsens the prognosis, [5,7,8] but objective measure of nutritional status, that is weight-for-age, has not been determined for this study and will be presented in a separate publication.

We did not collect data on *Haemophilus influenzae* type b (Hib) vaccine as this was not available when the CLHP developed and introduced the Inpatient Pneumonia Recording Forms in October 2000. Hib vaccine did not become available until 2002 and was limited to children who had not received their third DPT dose at the time of vaccine introduction. The data collected for this study only went up to 30 June 2003. This is a limitation of the study. The scale-up of Hib vaccine since 2002 and the inclusion of the 13-valent PCV into the Malawi EPI schedule since November 2011 is likely to further reduce the incidence and CFR of pneumonia in young children irrespective of HIV status. [27,28]

Those that received antibiotics prior to admission overall had worse outcomes. Those that attended and received an antibiotic at a primary care facility prior to admission to the district hospital had more severe disease, and severity of disease is strongly associated with outcome. This is mostly due to delayed presentation at primary care level. The fact that they were referred means they had a severe classification and severe classification is likely if they present late to a primary care facility and hence the poor outcome. This is the most likely explanation of the loss of significance of this factor in the multivariate analysis. We were also not able to determine other related issues of access to care or distance from facilities, or other socio-economic or behavioural factors that also determine use of care facilities and duration of symptoms before presentation.

Previous studies have noted major deficiencies with quality of care at the district hospital setting in many resource-limited settings. [29–31] Quality of care along with ready availability of effective therapy such as antibiotics and oxygen have an impact on outcome. [32] Efforts were made to minimise the impact of these important potential confounders on outcomes in this study population. This study was undertaken following training of health workers on standard case management as well as ensuring provision of an uninterrupted supply of antibiotics and oxygen therapy. However, despite a one-week training course, large numbers of children were still incorrectly classified. One issue that could not be addressed was the low staff numbers and the high turnover in all settings. [17]

It was noted that the provision of oxygen therapy, intravenous fluids or blood transfusion were all independently associated with a significantly higher CFR. The use of these therapies are all likely indicators of the severity of pneumonia and the presence of co-morbidities. Severity of hypoxaemia in children with pneumonia is associated with an increased risk of death. [33] There is clear evidence that oxygen therapy improves outcome in children with severe pneumonia, and that the routine use of oximetry improves effectiveness of oxygen therapy as it is more sensitive and specific for identifying hypoxaemic children than clinical indicators. [34] It is likely that in our study, the most hypoxaemic children were selected for oxygen therapy as pulse oximetry was not routinely available in this study and clinical indicators were relied upon. This is supported by the observation that mortality was higher in those that received oxygen, which reflects severity rather than impact of oxygen. The poor outcome in those receiving oxygen also suggests that PcP was not uncommon in this cohort. [32]

This study has identified several co-morbidities such as malnutrition and severe anaemia for which prevention is called for to improve the health of these vulnerable children. It also identified a number of potentially modifiable aspects of care where adjustments to the implementation of SCM are indicated. These include enhancing correct classification of the severity of the disease, the use of correct antibiotics according to standard case management, more extensive availability and use of oxygen together with oximetry to guide its use, universal testing for HIV and more routine use of simple laboratory tests to detect malaria and anaemia. To improve the outcome of these children with pneumonia, structural improvements are needed to address these needs. In addition, more comprehensive training to deal with co-morbid conditions might improve the quality of care. Further research is needed to investigate the process of transfer to hospital through the primary services and to demonstrate how laboratory services could be more efficiently used.

In conclusion, pneumonia-related mortality is common in Malawian infants and children cared for in the routine care hospital setting. Reducing mortality might be achieved through improved assessment, more rigorous application of standard case management and more accurate identification and effective management of co-morbidities.

## Supporting Information

S1 Data Excel Dataset of variables used in study. (XLSX)
